# Evaluation of the functional capacity and quality of life of children and adolescents during and after cancer treatment

**DOI:** 10.1590/1984-0462/2022/40/2020127

**Published:** 2021-09-01

**Authors:** Bruna Kuhn, Luciane Dalcanale Moussalle, Janice Luisa Lukrafka, Giana Berleze Penna, Abelardo de Oliveira Soares

**Affiliations:** a Universidade Federal de Ciências da Saúde de Porto Alegre, Porto Alegre, RS, Brazil.; b Universidade Federal do Rio Grande do Sul, Porto Alegre, RS, Brazil.

**Keywords:** Neoplasms, Pediatrics, Physical therapy, Walk test, Quality of life, Neoplasias, Pediatria, Fisioterapia, Teste de caminhada, Qualidade de vida

## Abstract

**Objective:**

To evaluate the functional capacity and quality of life of children and adolescents during cancer treatment and post-treatment.

**Methods:**

Cross-sectional study of patients during cancer treatment and post-treatment, assessed by the 6-minute walk test (6MWT) and the Pediatric Quality of Life ™ questionnaire (cancer module).

**Results:**

Sixty-five patients, aged 11.2±3.5 years, mostly males (50.8%) and white (90.8%), with high incidence of hematological cancers (81.5%) participated in the study. The performance in the 6MWT was 23.1% inferior comparing the mean predicted and achieved (584.3±5 and 447.7±78.6 m, respectively). The percentage difference between the predicted and achieved 6MWT, and the different phases of cancer treatment were significantly different between patients in treatment (73.7±12.8) and post-treatment (84.5±9.1). When grouped by the different diagnoses, it was possible to observe that the distance covered by the patients with leukemia showed results closer to that predicted (80.7±11.7). Regarding the health-related quality of life questionnaire (HRQL), the child’s perception (78.0±14.56) was better than that reported by their parents (72.4±17.74). However, when we compared HRQL with the 6MWT, there was no association between them (p=0.597).

**Conclusions:**

Children and adolescents undergoing cancer treatment or post-treatment showed a 23% deficit in functional capacity. In relation to HRQL results, children’s perception was higher than that of their parents.

## RESUMO

**Objetivo:**

Avaliar a capacidade funcional e a qualidade de vida de crianças e adolescentes em tratamento e pós-tratamento oncológico.

**Métodos:**

Estudo transversal de pacientes com câncer em tratamento ou pós-tratamento oncológico avaliados pelo teste de caminhada de 6 minutos (TC6) e pelo questionário Pediatric Quality of Life™ (módulo câncer).

**Resultados:**

Sessenta e cinco pacientes, com idade de 11,2±3,5 anos, sendo 50,8% do sexo masculino e 90,8% da raça branca, participaram do estudo, com mais incidência para os tumores hematológicos (81,5%). O desempenho no TC6 foi 23,1% inferior, quando comparadas as médias predita e realizada (584,3±5 m e 447,7±78,6 m, respectivamente). A diferença percentual entre o predito e o realizado do TC6 e as várias fases do tratamento oncológico foi significativa tanto entre os pacientes em tratamento (73,7±12,8%) como em pós-tratamento (84,5±9,1%). Quando separados os pacientes pelos diferentes diagnósticos, observou-se que a distância percorrida por aqueles com leucemia apresentou o resultado mais próximo do predito (80,7±11,7 m). No que diz respeito ao questionário sobre qualidade de vida relacionada à saúde (QVRS), a percepção da criança (78,0±14,5) foi melhor do que a relatada pelos pais (72,4±17,7). Mas, na comparação da QVRS com o TC6, não foi encontrada nenhuma associação (p=0,597).

**Conclusões:**

Crianças e adolescentes em tratamento ou pós-tratamento oncológico apresentaram déficit de 23% na capacidade funcional. Na comparação dos resultados da QRVS, a percepção das crianças foi superior à dos pais.

## INTRODUCTION

Childhood and adolescent cancer (0 to 19 years) consists of a set of diseases that have their own characteristics in relation to histological type, and leukemias (26%), lymphomas (14%) and tumors of the central nervous system (CNS - 13 %) are its most frequent forms.^1^ It is estimated that for each year of 2018 and 2019, there were 420,000 new cases in Brazil, of which about 12,500 were in this population and, more specifically, 1,300 in the southern region of the country.^2^

The disease results in different consequences, which can be linked to the disease itself, to the treatment or even to the post-treatment phase. It is common for unpleasant symptoms to appear during treatment, such as pain, nausea and fatigue, which are also related to the location of the tumor. In addition, the use of antineoplastic agents at an early age can lead to so-called late effects, such as decreased functional capacity.^3^ ^,^ ^4^ Intensive treatment, which includes surgery, chemotherapy, radiotherapy or a combination of these modalities, is often necessary in the search for a cure, but its adverse effects, such as serious infections, damage to certain organs (heart, lung, kidney, liver) and the decrease in bone mineral density, can also lead to a decrease in muscle strength and physical fitness, contributing to the patient’s weakness and worse health-related quality of life (HRQoL).^5^ ^,^ ^6^

Accordingly, with the growing number of survivors, the need for further studies is essential, with approaches on the assessment of functional capacity and HRQoL in pediatric cancer patients undergoing treatment. Understanding these processes is important for the development of appropriate interventions, which increase the level of functional capacity, since studies have already demonstrated a decrease of about 30%.

The main objective of this study was to assess the functional capacity and quality of life of children and adolescents under cancer treatment and post-treatment, and secondarily, to investigate the correlation between the self- and proxy-report of HRQoL and to compare the HRQoL and functional capacity and also the functional capacity of children and adolescents with solid tumors versus hematological cancers in the different phases of treatment.

## METHOD

Cross-sectional quantitative study, with a convenience sample, was carried out with children and adolescents diagnosed with cancer in inpatient or outpatient units at Hospital da Criança Santo Antônio (HCSA), in Porto Alegre (RS), a referral hospital for pediatric oncology, in January to September 2018. Children and adolescents who met the following criteria were selected: both sexes; age group 6 to 17 years; diagnosis of solid tumor, leukemia or lymphoma; having started radiotherapy and/or chemotherapy or surgery; or being on an outpatient basis — in the latter case, for children and adolescents who have completed treatment (post-treatment/follow-up). Excluded were the following patients: those with comorbidities that limited the performance of the tests, such as cognitive deficit; those in where there was restriction of the medical team due to lack of general clinical condition, such as active bleeding and blood count requiring blood transfusion; and those in palliative care.

The project was approved by the Human Research Ethics Committee of HCSA, under No. 1.958.759, in accordance with Resolution No. 466/2012. Patients whose guardians authorized the participation in the research, by signing an informed consent form, were also presented an approval form to obtain their acceptance and start of the study.

After the selection step, the patient registration form was completed, followed by the Pediatric Quality of Life (PedsQL™) Cancer Module 3.0 questionnaire, in the format of self-report and parental report (proxy-report), in the presence of the evaluator. It is composed of 27 items distributed in eight Likert-type scales, converted into numbers from zero to 100 points (0=100, 1=75, 2=50, 3=25 and 4=0), in which the higher scores indicate better HRQoL (less problems or symptoms).^7^

To assess functional capacity, the 6-minute walk test (6MWT) was applied, following the general criteria standardized by the American Thoracic Society (ATS). During the test, the modified Borg scale was also used, to determine the intensity of dyspnea and fatigue, with the patient himself pointing out his perception of effort during exercise. This exercise was performed on the 30-m course of a flat, covered corridor, with restricted flow of people, on the ground floor of the hospital. The total distance covered, as well as the total distance, was measured in meters, taking into account the Brazilian reference equations.^8^ ^,^ ^9^ ^,^ ^10^

As for the sample calculation, this was based on the study by Melo,^11^ considering an alpha of 5% and power of 80%, with an estimate of 48 patients to detect the difference between the intervention and control groups. Data were presented as mean ± standard deviation and median, for scalar data and absolute frequency, and percentage for categorical data.

The Shapiro-Wilk normality test was met only for the percentage between the predicted and achieved distance of the 6MWT. Comparisons of HRQoL between children and parents were carried out using the Wilcoxon test for paired samples. For comparisons between diagnoses and treatment phases, the Kruskal-Wallis and Mann-Whitney tests were applied, respectively.

To analyze the predicted percentage of the 6MWT, following the Brazilian equations, the tests used were Student’s t-test and analysis of variance (ANOVA). The correlations of the HRQoL domains between the self- and the proxy-report were determined using Spearman’s correlation coefficient. Significance level of p≤0.05 was used for all analyses, and the program Statistical Package for the Social Sciences (SPSS) 23.0 (SPSS Inc., United States) was used.

## RESULTS

Table 1 shows the characterization of the 65 patients who met the inclusion criteria in the study, with no exclusion or loss regarding the sample established by convenience. There was a higher incidence of hematological tumors (81.5%), and chemotherapy (98.5%) was the most common treatment.

**Table 1:** Characterization of cancer patients.

		Results n (%)
Age (n=65)*		11.2±3.5
Sex (n=65)		
	Male	33 (50.8)
Origin (n=65)		
	Porto Alegre and metropolitan region	38 (58.5)
	Countryside of Rio Grande do Sul	24 (36.9)
	Other Brazilian states	3 (4.6)
Skin color (n=65)		
	White	59 (90.7)
	Brown	4 (6.2)
	Black	2 (3.1)
BMI (n=65)		
	Underweight	19 (29.2)
	Normal weight	31 (47.7)
	Overweight and obesity	15 (23.1)
Diagnosis (n=65)		
	Leukemias	34 (52.3)
	Lymphomas	19 (29.2)
	Solid tumors	12 (18.5)
Chemotherapy protocol (n=64)		
	BFM 2002	23 (36)
	LHBRA 2015	7 (11)
	BFM 1995	5 (7.9)
	Brazilian protocol for Ewing family of tumors, non-metastatic	4 (6.2)
	GBTLI 2009	3 (4.6)
	Others	22 (34.3)
Radiotherapy protocol (n=2)		
	Radiotherapy	2 (3%)
Phase of hematological cancer treatment (n=53)		
	Initial	17 (32)
	Maintenance	18 (34)
	Follow-up	18 (34)
Phase of solid tumor treatment (n=12)*		
	Weeks of treatment	3.8±2.6

* Data expressed as mean±standard deviation; BMI: body mass index^31^; BFM: Berlin-Frankfurt-Münster European Group; GBTLI: Brazilian Group for the Treatment of Childhood Leukemia; LHBRA: Brazilian Hodgkin’s lymphoma.

**Figure 1:**
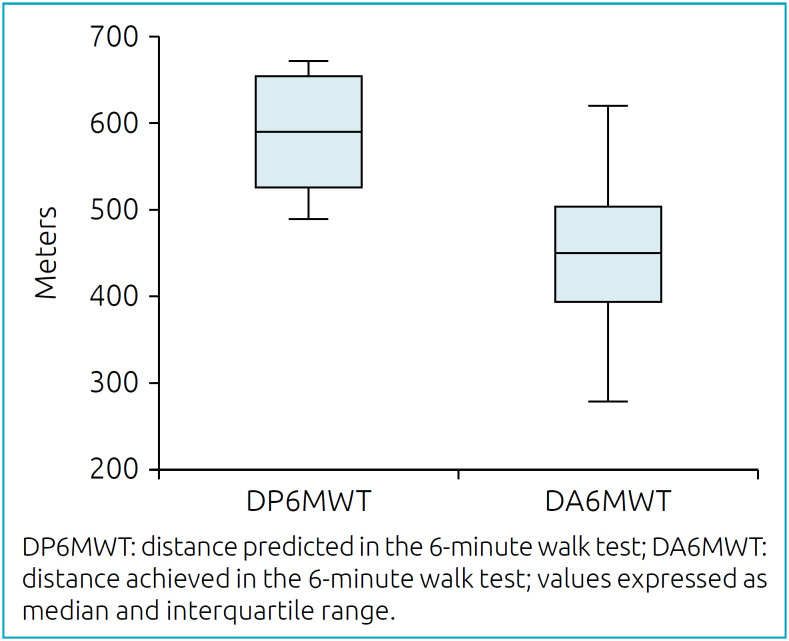
Distribution of the distance covered in the 6-minute walk test*.

Figure 1 shows the values predicted and achieved in the 6MWT. According to the reference equation, the difference between predicted and achieved was 23.1% (584.3±58 and 447.7±78.6 m, respectively), with a significant difference, p<0.001 [95% confidence interval— 95% CI (-26.3 to -19.9)]. No patient used supplemental oxygen, and there was no significant drop in SpO_2_ (greater than 4% from baseline) at the end of the 6MWT. The intensity of perception of leg fatigue and dyspnea were classified as mild by the modified Borg scale.

**Figure 2:**
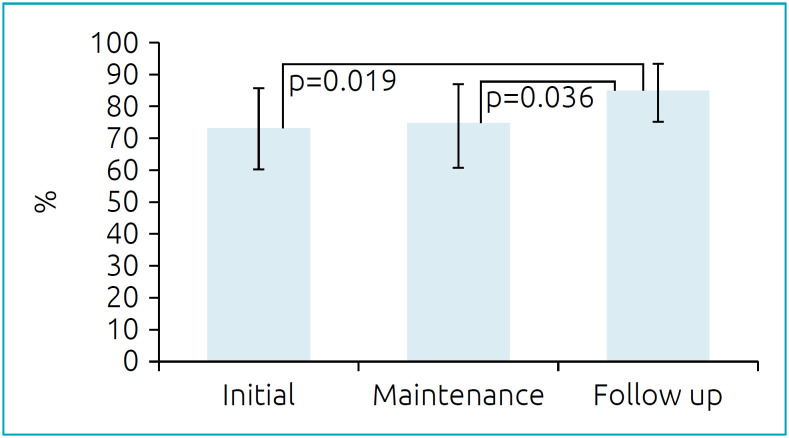
Percentage of distance covered in the 6-minute walk test in the different phases of chemotherapy.

Figure 2 shows the percentage difference between the predicted and the achieved distance in 6MWT in the different phases of chemotherapy, being statistically significant (p=0.003) when we grouped the patients under treatment (73.7±12.8%) and after treatment (84.5±9.1%). When comparing the percentage of the distance covered and the different types of cancer diagnosis, there was a statistically significant difference (p=0.012), with the distance achieved closest to that predicted in patients with leukemia (80.7±11.7), followed by patients with lymphomas (71.6±12.2) and solid tumors (64.2±13.9).

The HRQoL perceived by the child (78.0±14.5) was better than that reported by the parents (72.4±17.7), (p=0.001), and the results, separated by domains, are described in the Table 2. When correlated with HRQoL between self- and proxy-reported, a significant positive correlation was found: the higher the HRQoL in the general score of children’s perception, the better the parents’ perception was proportionally.

**Table 2:** Correlation of scores of the domains of health-related quality of life between the assessments made by the children (self-report) and those made by parents (proxy-report)*.

Domain	Median self-report (children)	Median proxy-report (parents)	r_s_	p-value
Pain	87.5 (50–100)	100 (62.5–100)	0.367	0.003
Nausea	80 (52.5–100)	80 (52.5–100)	0.677	<0.001
Anxiety before procedure	100 (50–100)	75 (50–100)	0.409	0.001
Anxiety in the face of treatment	100 (66.6–100)	66.7 (41.7–100)	0.345	0.005
Concern	83.3(50–100)	66.7 (41.7–100)	0.308	0.013
Cognitive difficulty	85 (62.5–97.5)	81.3 (65.6–97.5)	0.436	<0.001
Physical appearance	100 (75–100)	83.4 (66.7–100)	0.594	<0.001
Communication	91.7 (75–100)	83.4(66.7–100)	0.497	<0.001

* Data expressed as median (25–75% interquartile range); r_s_: Spearman correlation.

When analyzing the distance covered in the 6MWT and HRQoL, we did not find any statistically significant data in any of the domains or in the general assessment (p=0.080). When comparing HRQoL with the different types of cancer diagnosis, there was no statistically significant difference (p=0.957), nor between the phases of treatment (p=0.597).

## DISCUSSION

Worldwide and Brazilian epidemiological data on the incidence of pediatric cancers are in agreement with this study, showing a profile of predominance of males, whites and hematological cancers, with acute lymphoid leukemia (ALL) being the most common type in most populations, including that of Brazil, corresponding to between 25 and 35% of all types. ^2^ ^,^ ^12^

The diverse and intense therapies in childhood cancer, although increasingly specific, still have complications. It is known that there are numerous characteristics that influence motor function, and chemotherapy may result in anemia, decreased oxygen transport to the muscles and reduced muscle function. In addition, the use of some drugs, such as vincristine, can result in peripheral neuropathy with muscle weakness in the lower and upper extremities. These limitations, which often arise during treatment, can contribute to a sedentary lifestyle, with induction of inactivity, with a higher risk of obesity, cardiovascular disease, reduced muscle strength and, consequently, decreased HRQoL. ^13^ ^–^ ^16^

The 6MWT provides indicators of functional capacity, through the distance covered. In our study, patients covered 447.7±78.6 m, a lower index when compared to other studies conducted with the same population, which obtained values of 494.6 (95%CI 456.2–533.0) and 484.56±65.61. It is noteworthy that the lower result obtained can be explained by the fact that the patients from the outpatient clinic had only recently started physical therapy follow-up.^17^ ^,^ ^18^

In the study by Wallek et al.,^19^ the difference found between the 6MWT of the intervention group (with supervised exercises) and that of the control group (without exercises) in reducing physical performance was evident. Therefore, exercise programs implemented by physical therapists are important for this population, and accordingly, physiotherapy appears as a mechanism for training muscle function, seeking to optimize joint mobility to perform daily activities.^17^

Regarding the percentage of the 6MWT and the different phases of chemotherapy treatment, the data found here are consistent with those of published studies that demonstrate worse physical performance when comparing individuals undergoing treatment with healthy individuals, and their deficits may persist for years after treatment.^20^ ^,^ ^21^

In the study by Hooke et al.,^22^ the distance covered in the 6MWT was observed in the first three weeks after a new diagnosis, identifying inferior physical performance when compared to that of healthy individuals. Thus, as the treatment progresses, physical performance may remain unchanged and remain below normal, due to the effects of the treatment.^23^

As for the type of diagnosis, the mean of the 6MWT that was farthest from the predicted was seen in solid tumors. Hoffman^13^ also reported this finding, associating it mainly with invasive CNS surgery. In addition, another study described the implication of radiation as the main risk factor for poor results in physical performance, which we did not find in our study, as only one patient had undergone radiotherapy.^24^

Regarding HRQoL, a positive correlation was observed, confirming some studies that showed agreement between the reports, especially for diagnosed children who were up to 5 years old and who demand more care from parents or guardians.^25^ Thus, it is possible to infer that psychological factors or emotional ones, such as the anxiety and worry domains, are seen differently between parents and children. For this reason, children in the younger age group, who are quite ill or cognitively delayed, may have their reports replaced by those of their parents.^26^

Although there are no studies to date that correlate HRQoL with objective tests of functional capacity such as the 6MWT, there is a correlation with the subscale of functionality of the PedsQL. Fatigue is more commonly related to HRQoL, being considered a predictor of the worst score in this regard, especially in hospitalized patients,^27^ ^,^ ^28^ but our results demonstrated that there was no correlation with performance in the 6MWT, allowing children to have good performance in the 6MWT, even reporting fatigue in the HRQoL. It is noteworthy that children undergoing treatment with cardiotoxic drugs, such as anthracycline, have impaired HRQoL and may perform poorly on the 6MWT due to the physical restrictions imposed and tiredness during chemotherapy.^29^ ^,^ ^30^

As limitations of the study, we can identify the heterogeneity of the sample profile added to the diversity of chemotherapy protocols, which influences the extrapolation of data to the population studied.

In conclusion, the 6MWT is low-cost, reliable, feasible and valid for measuring physical performance, and can be easily used in pediatric cancer patients. It should be noted that children and adolescents undergoing cancer treatment are at high risk for deficits in functional capacity during and after cancer treatment. Therefore, more research is needed so that, in addition to identifying this deficit, it is possible to develop effective interventions, clearly demonstrating the benefits of physical activity, for the continuous improvement of health and HRQoL during cancer treatment, as well as patient survival.
